# Food insecurity and perinatal depression among pregnant women in BUNMAP cohort in Ethiopia: a structural equation modelling

**DOI:** 10.1017/S1368980024000855

**Published:** 2024-04-12

**Authors:** Abera Biratu, Atalay Alem, Girmay Medhin, Seifu Hagos Gebreyesus

**Affiliations:** 1 Department of Nursing, School of Health Sciences, Goba Referral Hospital, Madda Walabu University, Bale Goba, Ethiopia; 2 Departments of Psychiatry, School of Medicine, College of Health Science, Addis Ababa University, Addis Ababa, Ethiopia; 3 Aklilu Lema Institute of Pathology, Addis Ababa University, Addis Ababa, Ethiopia; 4 Department of Nutrition and Dietetics, School of Public Health, College of Health Sciences, Addis Ababa University, Addis Ababa, Ethiopia

**Keywords:** Food insecurity, Perinatal depression, Mediation, Rural Ethiopia

## Abstract

**Objective:**

To assess the effect of food insecurity on perinatal depression in rural Ethiopia.

**Design:**

We used a prospective cohort in which food insecurity was considered as primary exposure and perinatal depression as an outcome. Food insecurity at baseline (in the period of 8–24 weeks of pregnancy) was measured using the Household Food Insecurity Access Scale (HFIAS), and perinatal depression at follow-up (in 32–36 weeks of pregnancy) was measured using a Patient Health Questionnaire (PHQ-9). We used multivariable regression to assess the effect of food insecurity on the prevalence of perinatal depression. We explored food insecurity’s direct and indirect impacts on perinatal depression using structural equation modelling (SEM).

**Setting:**

This paper used data from the Butajira Nutrition, Mental Health and Pregnancy (BUNMAP) cohort established under the Butajira Health and Demographic Surveillance Site (BHDSS).

**Participants:**

Seven hundred and fifty-five pregnant women.

**Results:**

Among the study participants, 50 % were food-insecure, and about one-third were depressed at 32–36 follow-up. In SEM, higher values of baseline food insecurity, depressive symptoms and state–trait anxiety (STA) were positively and significantly associated with perinatal depression. The direct impact of food insecurity on perinatal depression accounts for 42 % of the total effect, and the rest accounted for the indirect effect through baseline depression (42 %) and STA (16 %).

**Conclusion:**

The significant effect of food insecurity at baseline on perinatal depression and the indirect effect of baseline food insecurity through baseline anxiety and depression in the current study implies the importance of tailored interventions for pregnant women that consider food insecurity and psychosocial problems.

Compared to men, women experience depression more frequently^(1)^, and it can manifest among women before, during, or after pregnancy and as well as recur and disappear throughout a woman’s lifetime^(2)^. Worldwide, the prevalence of perinatal depression during pregnancy ranges from 15 % to 65 %^(3)^, and in Ethiopia, it had a pooled prevalence of 21·28 %^(4)^. Perinatal depression can negatively affect the mother and the fetus^(3)^. It makes women less capable of taking care of themselves, less capable of providing care and more prone to morbidity from other causes^(5,6)^. Furthermore, it causes malnutrition, subpar physical and cognitive growth, and increased sickness in the offspring^(7)^. Some studies identified perinatal depression as a risk factor for low fetal birth weight and premature delivery^(3)^.

Causes of depression across life are usually complex and include psychosocial, environmental, biological and genetic factors^(8)^. The social determinants of mental health, or conditions in which people were born, grew, lived, and aged, profoundly impact mental health and mortality from other causes^(9)^. Social determinants of health can affect health through structuring the distribution of unmet health-related social needs for individuals^(10)^. Food insecurity is one of the common unmet social needs in Ethiopia. Even though poverty is declining, over 22 million Ethiopians live below the national poverty line^(11)^, indirectly demonstrating the prevalence of food insecurity. On the other hand, the patterns of income inequality as assessed by Gini coefficients show an upward tendency from 0·29 in 1995 to 0·30 in 2010/2011 and growing to 0·33 in 2015/2016^(11)^, which shows that the severity of food insecurity is being exacerbated in Ethiopia^(12)^.

Food insecurity, *‘a situation that exists when people lack secure access to sufficient amounts of safe and nutritious food for normal growth and development and an active and healthy life’* ^(13)^, is a primary concern globally, affecting about two billion people and increasing their vulnerability to malnutrition and poor health^(14)^. In 2016, household food insecurity in Ethiopia was estimated at 20·5 %, disproportionately affecting rural households compared to urban households on all indicators except calorie deficiency^(15)^. Women of reproductive age experience food insecurity more frequently^(16)^, particularly during pregnancy and lactation^(17,18)^. Research indicates that food insecurity can result in inadequate dietary intake and malnourishment^(16)^, placing people at risk for both poor physical and mental health outcomes^(19)^. Pregnant women who are food-insecure are more likely to have psychosocial issues like stress, sadness, anxiety and intimate partner violence (IPV)^(20–22)^.

Food-insecure pregnant women are more likely to experience IPV^(20)^. Using multivariable random effects models, Meyer *et al.* found a link between food insecurity and a higher risk of experiencing any kind of IPV^(23)^. According to Gelaye *et al.* ^(24)^, IPV is a significant risk factor for prenatal depression and is extremely common in low and middle-income countries (LMIC). In another comprehensive review and meta-analysis, IPV raised depressive symptoms and increased the risk of major depressive disorder (MDD) by 1·5 to 2 and 2 to 3 times, respectively^(25)^. After controlling for other factors, Navarette *et al.*’s study found that, in comparison to women who had never experienced IPV, IPV raised the probability of prenatal anxiety by 5·9 times and depression by 3·5 times, respectively^(26)^. When food insecurity and IPV coexist, pregnant women experience higher levels of stress.

Insecurity over food can cause psychological stress on its own. Cohen *et al.* view stress as a collection of constructs representing stages in a process wherein environmental demands that tax or exceed an organism’s capacity for adaptation result in biological, behavioural and psychological reactions that may increase an individual’s risk of illness^(27)^. When food insecurity and other psychosocial issues are combined with pregnancy, it becomes a much more demanding event, and IPV can exacerbate the sense of stress that comes with being pregnant. Excessive and prolonged stress during pregnancy can lead to an imbalance in the neural circuits that support mood, anxiety and cognitive functions, which can influence how those behaviours and behavioural states manifest^(28)^. The impact of such chronic changes and subsequent behaviour can have negative consequences^(29)^. According to some studies, the perception of higher-than-normal stress during pregnancy is linked to perinatal anxiety and depression^(30)^. Gokoel *et al.* discovered a significant link between high perceived stress and probable depression during pregnancy in a prospective cohort study of pregnant women^(31)^. Long-term anxiety can result in severe anxiety and mood symptoms, particularly depression^(32)^.

Lastly, there is substantial evidence from both high-income countries (HIC) and LMIC for a significant relationship between food insecurity and perinatal depression^(17,18,33)^. In a cross-sectional study in South Africa, higher odds of depressive symptoms were reported among food-insecure pregnant women^(17)^. In Ethiopia, food-insecure women were five times more likely to be depressed than food-secure women^(18)^.

However, the majority of LMIC studies were cross-sectional. Aside from that, the mechanism by which food insecurity affects perinatal depression has not been studied in pregnant women. Furthermore, no study has found that psychosocial factors such as IPV, perceived stress and anxiety play a role in mediating the relationship between food insecurity and perinatal depression. Food insecurity, IPV, perceived stress and depression are frequently seen in the same patient. Understanding the shared and unique effects of food insecurity, IPV, perceived stress and anxiety on depressive symptoms during pregnancy will have significant treatment implications. The current study sought to test the hypothesis that IPV, perceived stress and anxiety act as mediators between food insecurity and perinatal depression.

## Methods and subject

### Study design and setting

We conducted a prospective cohort study nested within the Butajira Nutrition, Mental Health and Pregnancy (BUNMAP) cohort, established in October 2017 and followed up until November 2020 in rural Ethiopia^(34)^. The BUNMAP cohort was established under the Butajira Health and Demographic Surveillance Site (BHDSS), which consists of nine rural and one urban administrative sub-districts representing the lowland, midland and highland agro-ecological settings^(35)^. Requirements of BUNMAP during its establishment were (a) pregnant women who have resided in the study area for at least 6 months, (b) 8–24 weeks of pregnancy and (c) willingness to participate in the study.

#### Study design

A prospective cohort study nested within the BUNMAP cohort study^(34)^.

#### Sample and sampling procedures

A cohort consisting of all pregnant women from the HDSS study population was recruited during the study period. Data were collected from all pregnant women (*n* 776) fulfilling the inclusion criteria. The Butajira HDSS monitors quarterly events, including pregnancy in the ten kebeles. However, health extension workers record pregnancies between those times. The HDSS enumerators make a connection to ensure accurate data. Using this system, we have used the list of pregnant women identified to recruit and enrol into the cohort.

### Data collection

Ten-day training was given to the data collectors and supervisors on specific modules and procedures to be applied for the data collection. Pregnant women within the study area were identified by data collectors who went house to house to interview every woman about her pregnancy status. Based on the report, women suspected to be pregnant had an appointment at the nearest health facility for further eligibility assessment. They were provided adequate information about the study when they arrived at the health centre on the appointment date. They were asked to consent if they volunteered to participate in the study.

After that, they were assessed for eligibility using an ultrasonography examination to determine their gestational age. If they were eligible, anthropometric measurements (height, weight and mid-arm circumference), blood pressure, finger-prick (for Hb) and a vein puncture (to withdraw 5 ml of blood for micronutrient analysis) were done at the health facility.

Within the same week, between 8 and 24 weeks of pregnancy, Time 1 (T1), trained data collectors went to the homes of study participants, and they collected data on the demography and economic status of the women, food insecurity and psychosocial factors such as stress, depression, anxiety and IPV. The data were collected electronically on tablets using Open Data Kit (ODK) software. The collected data were submitted to a secure server via an Internet connection. Within the study period, follow-up assessments were repeated between 32 and 36 weeks of gestation, Time 2 (T2).

### Exposure variable: household food insecurity

We used the Household Food Insecurity Access Scale (HFIAS) to measure the household-level magnitude of food insecurity. It was developed by the USAID-funded Food and Nutrition Technical Assistance II Project (FANTA) in collaboration with Tufts and Cornell Universities^(36)^. The HFIAS is a nine-item scale with self-reported items using a recall period of 4 weeks and response categories relating to the occurrence and frequency of occurrence^(37)^. First, the respondents were asked if they encountered the condition (yes or no) and, if so, how frequently (rarely, occasionally or often) they encountered it. A continuous or categorical indicator of food insecurity can be used from the obtained response. Each of the nine occurrence frequency questions is scored 0–3, and the scores are totalled while computing HFIAS as a continuous measure. The degree of food insecurity is indicated by the overall HFIAS score, which runs from 0 to 27. When taken as a categorical variable, households are categorised as food-secure, mildly food-insecure, moderately food-insecure and severely food-insecure^(37)^. The HFIAS captures three domains of food insecurity experience: anxiety and uncertainty about supply, insufficient quality, and insufficient intake and physical consequences^(36)^. The reliability coefficient, Cronbach’s α for the HFIAS total score in this study, was 0·79.

### Mediating variables

The measures of the following mediating variables were conducted at T1.

### Intimate partner violence

The Hurt, Insult, Threaten, Scream (HITS) screening tool, which was developed by Sherin *et al.* ^(38)^, was used to measure IPV. This tool utilises four questions asking how often the partner of the respondent does the following: ‘physically hurt you’, ‘insult or talk down to you’, ‘threaten you with harm’ and ‘scream or curse at you’. Each item is scored with a 1–5 Likert scale for the frequency of the behaviour, with one being ‘never’ and five being ‘frequently’. The sum score of the responses ranges from 4 to 20, with higher scores indicating higher interpersonal violence^(38)^. In this study, the reliability coefficient, Cronbach’s α for the total HITS items score, was 0·71.

### Anxiety

Pregnancy-related anxiety (PRA), which is conceptualised as a woman’s fear about her baby’s health, her health, and labour and delivery^(39)^, was assessed using a twelve-item pregnancy-related anxiety questionnaire. The reliability coefficient, Cronbach’s α for total score pregnancy-related anxiety questionnaire items, was 0·93 for this study. Another questionnaire used to measure anxiety was the State-Trait Anxiety Inventory (STAI-6), which was developed to provide reliable, relatively brief, self-report scales for assessing state and trait anxiety (STA) in research and clinical practice. It is a commonly used measure of trait and state anxiety^(39,40)^. It has acceptable reliability and produces scores similar to those made with full-form across subject groups, manifesting normal and raised anxiety levels^(40)^. In this study, the reliability coefficient, Cronbach’s α for the STAI-6 total score, was 0·79. Regarding the factorial and the validity of STAI-6 of this study, the Kaiser–Mayer–Olkin (KMO) test of sampling adequacy value was 0·76. The Bartlett’s sphericity test value was <0·001. The determinant score (0·101) found no multicollinearity issues in the STAI-6 item score. The result of factor analysis showed a rotated factor solution for STAI-6 contained two factors with eigenvalues >1·0, which accounted for 58 % of the variance component. Items 2, 3 and 6 strongly correlated with factor 1, that is, the presence of anxiety, and for all these variables, the correlation between the items and the underlying construct was >0·70. Items 1, 4 and 5 have loaded on factor 2, that is, absence of anxiety, and item 4 is most strongly associated with the underlying construct with a correlation of 0·71. The two factors of confirmatory factor analysis of STAI-6 indicated the best-fit of comparative fit index (CFI) (1·000), Tucker–Lewis index (TLI) (1·000), root mean square error of approximation (RMSEA) (0·000, 90 % CI: 0·000, 0·072) and standard root mean square residual (SRMR) (0·007).

### Maternity Social Support Scale

Maternal social support was measured using the Maternity Social Support Scale (MSSS). The scale contains six items. Each item has response options on a five-point Likert scale and a total possible score of 30. This study’s reliability coefficient, Cronbach’s α for MSSS total score, was 0·68.

### Stress

History of life events experienced within the last 1 year (12 months) was assessed using the List of Threatening Experiences Questionnaire (LTE). The LTE questions were based on twelve yes/no questions about events that may have occurred within the past 12 months. The total score of LTE ranges from 0 (no LTE experienced) to 12 (all LTE experienced). This study’s reliability coefficient, Cronbach’s α for LTE total score, was 0·62.

The Perceived Stress Scale (PSS) was initially developed by Cohen *et al.* and was utilised to measure stress symptoms^(41)^. This scale includes ten questions assessing the frequency of specific feelings and thoughts over the last month, using a five-point Likert scale rate of 1–5, with one being ‘never’ and five ‘almost always’. The sum score ranges from 10 to 50, with higher scores indicating more perceived stress. This study’s reliability coefficient, Cronbach’s α for PSS total score, was 0·83.

### Covariates

A range of sociodemographic and health data of the mother’s education, religion, marital status, occupation, and partner’s education and occupation were collected using a questionnaire adapted from the Ethiopian Demographic and Health Survey and added questions based on relevant literature^(42)^. We also collected household data such as food and non-food consumption expenditure, production and income, ownership and size of land, type of house and construction materials, availability of radio, television, telephone, bed, chair, and other household items, possession of domestic animals, and sanitation facility and source of water were also collected. Using twenty-six wealth indexes that were modified from the central statistical agency (CSA)^(42)^, wealth index quintiles were computed using principal component analysis. Using principal component analysis, the data were sorted from poorest to wealthiest, with the twenty, forty, sixty, eighty, and 100 percentiles allocated to the poorest, poorer, medium, richer, and richest, respectively. All covariates were assessed at T1.

### Hb as a measure of anaemia

Anaemia was assessed at baseline by measuring Hb in erythrocytes from finger-prick blood samples using a Hemo-Cue (Hb-201) instrument. Pregnant women with an Hb level below 11 g/dl were considered anaemic.

### Anthropometry assessment

Mid-upper arm circumference (MUAC) was used to estimate maternal nutritional status at baseline. It was measured three times at the midpoint between the tip of the shoulder and the elbow of the left upper arm using inelastic adult MUAC tape. The average of three MUAC measurements was calculated and then categorised as normal or low MUAC.

### Data quality control

Data collectors received training on how to approach the participants and collect data to minimise technical and observer bias. The mean of the two measures of MUAC, weight and height were taken to ensure accuracy. The collection of specimens and laboratory procedures were carried out following standard operating procedures. A pretest was made on 5 % of the total sample size of the study population in an adjacent study setting. The data collectors double-checked a questionnaire for accuracy before submitting it to the supervisor for approval. The supervisors assessed the quality of the data before its transfer to an EPHI central database. The Strengthening the Reporting of Observational Studies in Epidemiology (STROBE) guideline was followed in the reporting of our study^(43)^.

### Outcome assessment

Depression symptoms were measured using the Patient Health Questionnaire-9 (PHQ-9) at T1 and repeated at T2. The PHQ-9 consists of nine questions with a 2-week memory interval. The questions ask participants how frequently they have had depressed symptoms, and the possible answers are 0, ‘not at all’, 1, ‘several days’, 2, ‘More than half the days’, and 3, ‘nearly every day’. In a previous study, the PHQ-9 was validated in Amharic in the study area’s primary healthcare settings^(44)^. A score of 5 or higher during pregnancy indicated the presence of perinatal depression symptoms. The reliability coefficient, Cronbach’s α for PHQ-9 total score, was 0·80 with CI (0·769, 0·823) in this study.

### Data processing and analysis

Data management and statistical analysis were performed using Stata version 16.0 (StataCorp LLC).

The analysis was based on BUNMAP cohort study participants who responded to the questionnaire during the baseline assessment for mediating variables and had complete data on depressive symptoms at the follow-up assessment. Descriptive statistical summaries were presented as mean and standard deviation for continuous variables and as frequencies and percentages for categorical variables. The internal consistency of each scale was assessed using Cronbach’s α.

After examining the distribution of the food insecurity score at baseline and depressive symptoms score at T2, resulting in positively skewed histograms, logarithmic transformation was used before further analysis. Bivariate associations of the outcome variable with the exposure variable and other potentially confounding variables (sociodemographic, wealth indices variables, nutritional status and psychosocial variables) were assessed using *χ* ^2^ and *t* test as appropriate.

Before conducting the final analysis using structural equation modelling (SEM) with STATA SEM builder through multiple regression analysis, only statistically significant paths (*P* < 0·05) were used to build an initial path model. Besides this, we performed a mediation analysis for each of the proposed variables (STA, PRA, LTE, perceived stress, IPV, MSS and baseline PHQ-9) separately with the medsem command. Those variables found to be a mediator (i.e. their paths were statistically significant) were included in the initial model. SEM with maximum likelihood estimation was used to test pathways between food insecurity, potential mediators and perinatal depression. Then, we applied modification indices and evidence from the literature to modify model specifications. Non-significant paths were trimmed out from the model. We fit two SEM. The first one was based on the 520 respondents for whom depression scores were recorded at follow-up time, and food insecurity, depressive symptoms, STA, IPV and perceived stress scores were recorded at baseline. The second SEM was based on all 755 respondents, with missing values estimated by multiple imputations. We imputed 100 cases using the multivariate imputations by chained equations (MICE), with 1000 iterations^(45)^. We used bias-corrected bootstrapping and 1000 iterations to determine direct and indirect effects with 95 % CI of the relationship (i.e. paths that link risk factors and the outcome) between food insecurity and perinatal depression. Based on existing literature, a significance level of 0·05 (1–95 %) is considered for the p-value; therefore, if *P* < 0·05, the null hypothesis is rejected.

Model fit was assessed based on relative fit indices: the RMSEA good-fit statistic (RMSEA ≤ 0·08), the CFI and the TLI goodness-of-fit statistic (CFI ≥ 0·90 and TLI ≥ 0·90), SRMR well-fit statistic (SRMR < 0·05).

## Results

The baseline sociodemographic characteristics of the study participants are summarised in Table 1. Of the 776 eligible women enrolled in the cohort, 755 (97·2 %) were included in the analysis. The reason for excluding cohort members was missing psychological data. The mean age of the study respondent was 27·8. They were predominantly residents of rural areas (78·6 %), Gurage by ethnicity (68·6 %), Muslim by religion 631 (83·58 %), housewives (77·3) and married (99·6 %). Very few participants (20·2 %) were categorised under rich SES.

**Table 1:** Sociodemographic characteristics of the study population (*n* 755)

Maternal characteristics		*n*	%
Woman’s age*	Year	27·81	5·08
Residence	Urban	162	21·46
	Rural	593	78·56
Educational status	Primary (1–8)	353	46·75
	Secondary and above	69	9·14
	Read and write	55	7·28
	Not literate	278	36·82
Religion	Orthodox Christian	83	10·99
	Islam	631	83·58
	Protestant	41	5·43
Ethnicity	Gurage	518	68·61
	Silte	165	21·85
	Others	72	9·54
Occupational status	Farmer and housewife	78	10·33
	Housewife	583	77·22
	Merchant	64	8·48
	Other	30	3·97
Marital status	Currently married	752	99·60
Socio-economic status	Poorest	146	19·92
	Poor	147	20·05
	Middle	145	19·78
	Rich	148	20·19
	Richest	147	20·05

* Data are represented as mean and standard deviation.

The clinical status and food insecurity status of the participants are summarised in Table 2. The mean and standard deviation of MUAC, Hb, BMI and gestational age of the study participants at baseline were 24·71 (s d = 2·16), 13·08 (s d = 1·19), 15·51 (s d = 4·79) and 16·72 (s d = 4·49), respectively. Among the study participants (*n* 755) at baseline, 49·93 % were food-insecure. In terms of severity, eighty-seven (11·5 %) were mildly food-insecure, 254 (33·6 %) were moderately food-insecure and thirty-six (4·8 %) were severely food-insecure. Among those women assessed at T2 (*n* 521), a total of 165 (31·67 %) had high perinatal depressive symptoms (>5 on the PHQ-9).

**Table 2:** Clinical and food insecurity status of the study participants (*n* 755)

Characteristics		Mean	sd	*n*	(%)
MUAC		24·71	2·16		
Hb		13·08	1·19		
BMI		15·51	4·79		
GA		16·72	4·49		
Food insecurity score		2·65	3·59		
PHQ-9 score at T1		3·58	4·19		
PHQ-9 score at T2		3·50	4·15		
LTE		0·87	1·33		
Perceived stress		16·29	5·38		
State–trait anxiety		13·08	4·45		
PRA		25·58	10·34		
MSSS		21·81	4·60		
IPV		4·70	1·45		
Binary food insecurity status	Food-secure			378	50·07
	Food-insecure			377	49·93
Category of food insecurity	Food-secure			378	50·07
	Mildly food-insecure			87	11·52
	Moderately food-insecure			254	33·64
	Severely food-insecure			36	4·77
Perinatal depression	Not depressed			356	68·33
	Depressed			165	31·67

MUAC, mid-upper arm circumference ; GA, gestational age; PHQ-9, Patient Health Questionnaire; LTE, list of threatening experiences; MSS, Maternity Social Support Scale; IPV, intimate partner violence.

Supplementary Table 1 shows bivariate analysis between food insecurity and other variables. There was a significant difference between food-insecure and food-secure pregnant women in terms of residence, educational status, religion and ethnicity. Regarding socio-economic status, food insecurity is significantly associated with all categories. Pregnant women who were food-insecure compared with food-secure were more likely to have a higher mean LTE score (1·17 *v*. 0·57 *P* < 0·001), PRA score (26·76 *v*. 24·41, *P* = 0·002), STAI-6 score (13·84 *v*. 12·32, *P* < 0·001), PHQ-9 score at T1 (4·82 *v*. 2·50, *P* < 0·001) and PHQ9-score at T2 (4·69 *v*. 2·22, *P* < 0·001). No significant differences were observed between food-insecure and food-secure pregnant women regarding pregnant women’s age, occupation, marital status, MUAC, Hb, BMI, gestational age, perceived stress, MSS and IPV.

In multivariable regression models that adjusted for age, residence, education, SES, MUAC and BMI, food insecurity was significantly associated with a high score of perinatal depression (*β*, 0·27; 95 % CI, 0·173– 0·359; *P* < 0·001) (Table 3). This association was primarily seen among women who lived in moderately food-insecure households (*β*, 0·58; 95 % CI, 0·398, 0·771; *P* < .001). Although the strength of association was lowered in the final model after entering possible mediating variables, food insecurity remained significantly associated with perinatal depression (*β*, 0·10; 95 % CI, 0·008, 0·189; *P* = 0·033).

**Table 3:** Multivariable linear regression analysis to explore the association between food security and perinatal depression (*N* 755)

					Model I			Model II		
Characteristics		*β*	95 % CI	*P*	*β*	95 % CI	*P*	*β*	95 % CI	*P*
HFIAS score		0·26	0·176, 0·345	<0·001	0·27	0·173, 0·359	<0·001	0·10	0·008, 0·189	0·033
Binary of food insecurity		0·58	0·422, 0·743	<0·001	0·55	0·379, 0·723	<0·001	0·29	0·131, 0·457	<0·001
Category of food insecurity	Food-secure	Ref		<0·001	Ref			Ref		
	Mildly food-insecure	0·65	0·375, 0·931	<0·001	0·51	0·225, 0·791	<0·001	0·54	0·284, 0·800	<0·001
	Moderately food-insecure	0·58	0·411, 0·759	<0·001	0·58	0·398, 0·771	<0·001	0·22	0·038, 0·400	0·018
	Severely food-insecure	0·38	–0·050, 0·801	0·084	0·33	–0·100, 0·766	0·131	0·11	–0·277, 0·503	0·568
PHQ-9 at T1		0·46	0·384, 0·543	<0·001				0·37	0·278, 0·469	<0·001
Perceived stress		0·04	0·028, 0·056	<0·001				0·001	–0·018, 0·021	0·891
State–trait anxiety		0·06	0·047, 0·080	<0·001				0·04	0·015 0·062	0·001
IPV		0·10	0·044, 0·152	<0·001				−0·002	–0·054, 0·048	0·915

HFIAS, household food insecurity access scale; PHQ, Patient Health Questionnaire; IPV, intimate partner violence.

**Fig. 1:**
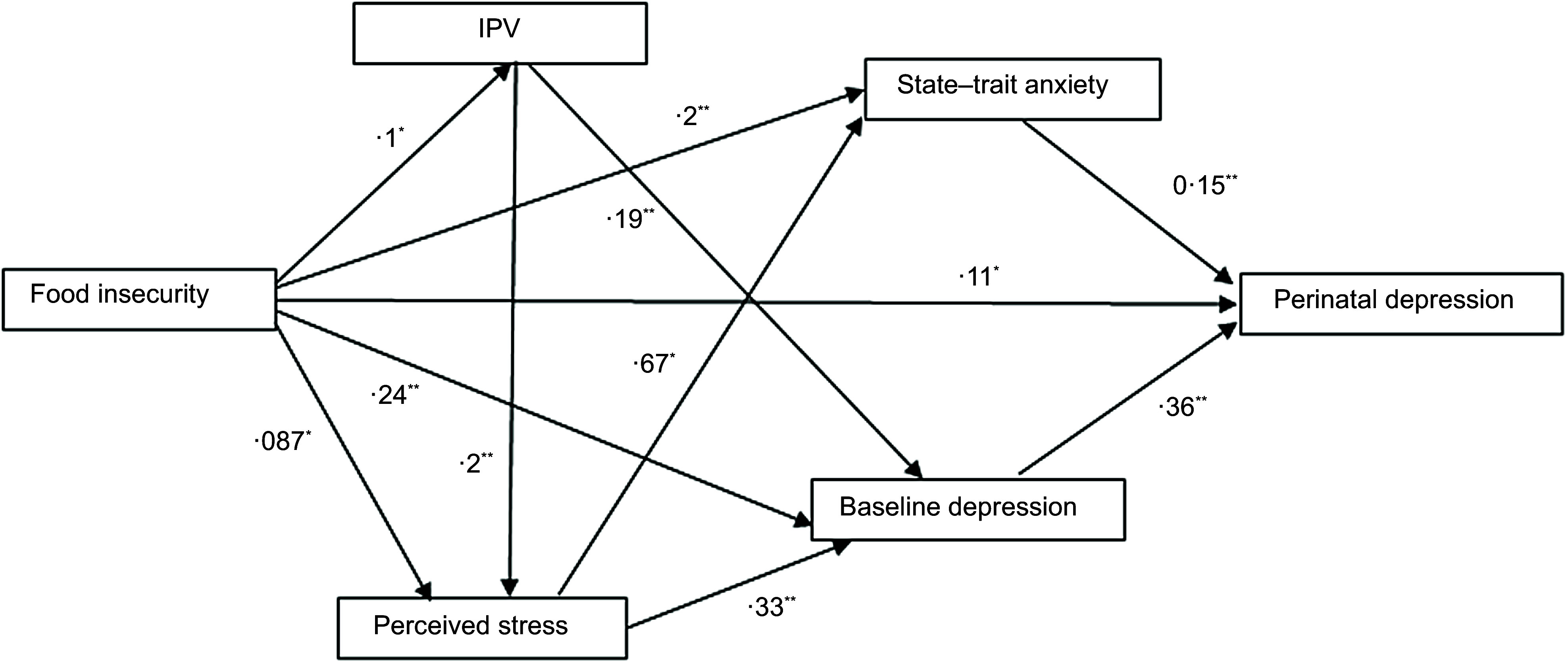
Structural equation model of the relationship between food insecurity and perinatal depressive symptoms (*n* 520). RMSEA = 0·010 (90 % CI = 0·000, 0·068); CFI = 1·000; TLI = 0·999; SRMR = 0·014. All relationships are significant at *P* < 0·05. * *P* value less than 0·05, ** *P* value less than 0·001 indicates significant path coefficients. IPV, intimate partner violence; RMSEA, root mean square error of approximation; CFI, comparative fit index; TLI, Tucker–Lewis index; SRMR, standard root mean square residual

**Fig. 2:**
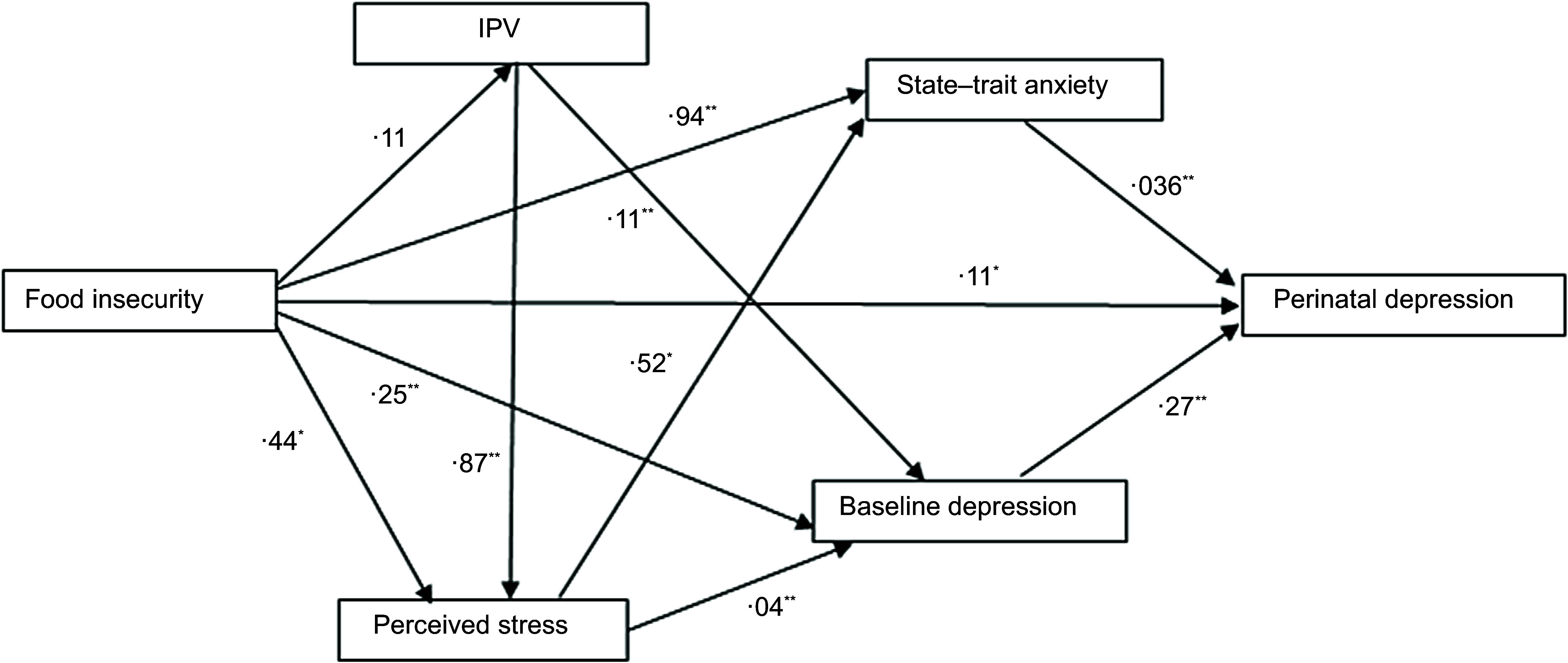
Structural equation model of the relationship between food insecurity and prenatal depressive symptoms (*n* 755). All relationships are significant at *P* < 0·05. * *P* value less than 0·05, ** *P* value less than 0·001 indicates significant path coefficients. IPV, intimate partner violence

Figures 1 and 2 illustrate the structural model for the association between food insecurity and perinatal depression, controlling for covariates. The initial model did not achieve acceptable goodness of fit across all metrics. Therefore, we used modification indices to add four paths to indicator variables in the model (Table 4). We removed the path between (a) perceived stress and perinatal depression and (b) IPV and perinatal depression, which were not statistically significant.

**Table 4.** Goodness-of-fit indices for each model modification

Model	RMSEA	90 % CI	CFI	TLI	SRMR
Initial model	0·390	0·361, 0·420	0·328	−0·680	0·205
Adding correlation between IPV and perceived stress	0·418	0·386, 0·451	0·358	−0·926	0·201
Adding correlation between IPV and baseline depression	0·447	0·411, 0·483	0·413	−1·200	0·185
Adding correlation between perceived stress and baseline depression	0·471	0·430, 0·514	0·510	−1·451	0·167
Adding correlation between perceived stress and state–trait anxiety	0·045	0·000, 0·108	0·997	0·978	0·014
Removing the correlation between perceived stress and perinatal depression	0·027	0·000, 0·083	0·998	0·992	0·014
Removing the correlation between IPV and perinatal depression	0·010	0·000, 0·068	1·000	0·999	0·014

RMSEA, root mean square error approximation; CFI, comparative fit index; TLI, Tucker–Lewis index; SRMR, standard root mean square residual; IPV, intimate partner violence.

After these modifications, the model with food insecurity as a continuous variable attained a good level of model fit, excluding missing values (Fig. 1) and replacing missing values with multiple imputations (Fig. 2).

We found a significant path coefficient between food insecurity and IPV (*β*, 0·11; s e, 0·05; *P* = 0·026), baseline depression (*β*, 0·05; s e, 0·02; *P* = 0·005), state-trait anxiety (*β*, 0·20; s e, 0·03; *P* < 0·001) and perceived stress (*β*, 0·087; s e, 0·04; *P* = 0·041). There was a significant association between food insecurity (*β*, 0·11; s e, 0·04; *P* = 0·007), state-trait anxiety (*β*, 0·15; s e, 0·04; *P* < 0·001) and baseline depression (*β*, 0·36; s e, 0·04; *P* < 0·001) with perinatal depression (see online supplementary material, Supplementary Table 2). Food insecurity had an indirect association with perinatal depression through state-trait anxiety (*β*, 0·04; 95 % CI, 0·014, 0·069; *P* < 0·001) and baseline depression (*β*, 0·11; 95 % CI, 0·072, 0·159; *P* < 0·001) (Table 5).

**Table 5.** Mediation path of the relationship between food insecurity and perinatal depression

Food insecurity	Effect	Bootstrap 95 % CI	*P* value	% Total effect of food insecurity on perinatal depression
The total effect of food insecurity on perinatal depression	0·26	0·178, 0·351	<0·001	
The total indirect effect of food insecurity on perinatal depression	0·15	0·104, 0·202	<0·001	58
The direct effect of food insecurity on perinatal depression	0·11	0·029, 0·194	0·001	42
Indirect paths				
Depressive symptoms at T1	0·11	0·072, 0·159	<0·001	42
State–trait anxiety	0·04	0·014, 0·069	<0·001	16

In mediation analysis, we found a significant direct effect between food insecurity and a high score of perinatal depression after adjusting for the indirect effects. The direct effect of food insecurity on perinatal depression accounted for 42 %, while its indirect effect was 58 %. The indirect impact of food insecurity on high scores of perinatal depressive symptoms was mediated through baseline depressive symptoms (42 %) and STA (16 %). (Table 5).

## Discussion

We aimed to assess the effect of food insecurity on perinatal depression and the mediating effect of IPV, perceived stress and anxiety on this association among pregnant women in rural Ethiopia. Half of the participants in this study were food-insecure at baseline, and about one-third of the women reported elevated levels of perinatal depressive symptoms at follow-up. In multiple regression, food insecurity significantly affected perinatal depressive symptoms. In the SEM analysis, food insecurity was significantly associated with perinatal depression both directly and indirectly, and elevated levels of anxiety and depression at T1 mediate the relationship when included in the association. Although IPV and perceived stress had no direct effect on perinatal depression and did not mediate the relationship between food insecurity and perinatal depression, they also played a significant role. IPV had an indirect impact on perinatal depression through perceived stress, state-trait anxiety and baseline depressive symptoms. Perceived stress also had an indirect effect on perinatal depression through state-trait anxiety and depressive symptoms at baseline.

In this study, SEM analysis elucidated a positive relationship between food insecurity and perinatal depression. Of the total effects of food insecurity on perinatal depressive symptoms, 42 % was shown to be over through a direct path. This finding agreed with previous studies conducted in LMIC among pregnant women^(17,18)^. Abrahams *et al.* assessed the relationship between food insecurity and perinatal depression and demonstrated that food insecurity was a strong predictor of perinatal depression^(17)^. There is also supporting evidence from HIC that food insecurity and perinatal depression are interconnected and called for intervention to improve food insecurity and perinatal depression concurrently^(46)^. Food insecurity also indirectly affects perinatal depression through anxiety.

More than half (58 %) of the effect of food insecurity on perinatal depression was mediated through baseline depressive symptoms and state-trait anxiety. Baseline depressive symptoms partially mediate 42 % of the indirect effect of food insecurity on perinatal depression. In this study, high depressive symptoms were present from baseline in nearly two-thirds of pregnant women reporting perinatal depression at T2. The contribution of food insecurity to prolonged depressive symptoms is paramount. Of those who reported continued depressive symptoms, 75 % reported food insecurity at the baseline. Once depression occurs in a person’s life, it is more likely to persist and recur later^(2)^. Similarly, in a prospective cohort study, the perinatal depression continued throughout the postnatal period in a significant portion of the population^(47,48)^. Such chronic depression was associated with poor pregnancy outcomes^(3)^. So, early detection and treatment of depressive symptoms during pregnancy are vital to decrease the burden of depression.

The level of state-trait anxiety was higher among food-insecure women, which significantly increased their depressive symptoms. Many studies reported that food insecurity is one of the crucial predictors of anxiety^(49,50)^. In a cross-sectional study involving 376 pregnant women, Heyningen *et al.* found that pregnant women experiencing food insecurity had a 2·6-fold increased risk of developing anxiety disorders compared to those not food-insecure^(51)^. In the current study, state-trait anxiety was also significantly associated with perinatal depression at T2. This finding was consistent with the previous study^(30)^. Xian *et al.* found that those pregnant women who presented with anxiety were 8·9 times more likely to have prenatal depression^(30)^. Moderate anxiety is typical for an individual in their life, but a constant state of anxiety can alter the functional ability of the women and can lead to depression^(32)^. More than 50 % of those women who develop depression are more likely to have a co-morbid anxiety disorder^(52)^. Perinatal depression, when coexisting with anxiety, is associated with a higher risk of suicide, prolonged illness duration and a greater likelihood of treatment non-response^(53)^. In the present study, the odds of reporting a positive response on item 9 of PHQ-9 are almost ten times more likely among those who scored above the mean STAI-6 score than those who reported below the mean at follow-up time.

In this study, food insecurity significantly predicts high levels of perceived stress, which aligns with the previous study^(21,22)^. Food insecurity was linked to 22 % higher levels of stress, according to Nikoonia *et al.*’s analysis, after controlling for the confounder effect in the final model^(21,22)^. In a cross-sectional study involving 421 Pakistani women of reproductive age, Zahid *et al.* found a substantial association between food insecurity and stress, with food-insecure women having 3·8 times higher odds of experiencing stress^(22)^. Contrary to what we hypothesised, high perceived stress did not mediate the relationship between food insecurity and perinatal depression. However, our finding shows that women with elevated perceived stress are more likely to have high levels of state-trait anxiety and high levels of depressive symptoms at baseline, and these pathways indirectly predict the probability of perinatal depression at T2. Chronic stress in life is a psychosocial factor that may induce long-lasting changes in gene expression in different neural structures^(54)^. Such changes are thought to be the possible causes of stress-related disorders such as anxiety and depression^(54)^. In a more recent cohort study with 1143 pregnant women, it was found that women with high levels of stress during the first two trimesters had nearly two times the likelihood of experiencing probable depression during the third trimester than women with low levels of stress^(31)^. In addition to policies and interventions to address food insecurity, it is also vital for early detection of different psychological problems during pregnancy that contribute to the development of perinatal depression. Transdiagnostic treatment, such as unified protocol, which is used to address common mental health issues like anxiety, depression and other emotional disorders, can help reduce the severity and outcomes of perinatal depression^(32)^.

Food insecurity has a significant effect on IPV. This finding is consistent with Hatcher *et al.*, who reported a direct and indirect relationship between food insecurity and IPV^(55)^. Baseline IPV score is directly related to baseline depression, but this relationship was not maintained in T2 with depressive symptoms. However, IPV indirectly predicted depressive symptoms at T2 through perceived stress, state-trait anxiety and baseline depression. Previous studies revealed that IPV is an important predictor of perinatal depression^(20,26)^. Navarette *et al.* claim that IPV increases the risk of depression during pregnancy and 6 months postpartum^(26)^. IPV may cause stressful situations that can stimulate the hypothalamic–pituitary–adrenal axis to generate hormones that arouse emotions^(56)^, and this may be possibly associated with depressive symptoms^(57)^. It is advised that more research be done to assess IPV prevention measures to lessen the impact of depression^(25)^.

More research is required to expand our findings and explore these mediation effects.

## Strengths and limitations of the study

The current study sought to determine the causal mechanisms between food insecurity and perinatal depressive symptoms using a prospective cohort strategy. Understanding this connection is necessary to develop efficient interventions to prevent perinatal depression and its detrimental consequences for women and their offspring. However, this study is not free of limitations. The first shortcoming is that there are many losses to follow up on even though we have tried to manage it with multiple imputations for the missing values. We also did not assess the direction of the relationship between food insecurity and perinatal depression. Lastly, the measures we used for pregnancy-related anxiety and STAI-6 were not validated or adapted in line with the context of the study setting. However, internal consistency and the factorial and construct validity of the tool were checked for this study.

## Conclusion

In this study, pregnant women who were food-insecure had significantly higher depressive symptoms than women who were food-secure. Baseline depressive symptoms and STA mediated 58 % of the effect of food insecurity on perinatal depression. IPV and perceived stress had an indirect impact on the development of perinatal depression. Interventions that address food insecurity and these psychosocial factors at an early stage of pregnancy would be needed to minimise the unwanted impact of food insecurity on perinatal depression. Programmes and policies should emphasise increasing livelihood options and implementing integrated mental health care for pregnant women, which can foster women’s physical and psychological well-being.

## Supporting information

Biratu et al. supplementary material Biratu et al. supplementary material

## Supplementary material

For supplementary material accompanying this paper visit https://doi.org/10.1017/S1368980024000855
