# Supplementary material for: Food insecurity and perinatal depression among pregnant women in BUNMAP cohort in Ethiopia: a structural equation modelling
Source: Public Health Nutr. 2024 Apr 12;27(1):e120. doi: 10.1017/S1368980024000855 (PMC11075105; doi:10.1017/S1368980024000855)
Supplement: Biratu et al. supplementary material [file S1368980024000855sup001.docx]

| Supplementary Table 1: Sociodemographic characteristics of food-secure compared with food-insecure pregnant women (n=755) | | | | |
| --- | --- | --- | --- | --- |
| **Characteristics** | | **Food secure (N=378)** | **Food insecure (N=377)** | **P-value** |
| Age, y | Mean (SD) | 27.66(5.01) | 27.97(5.16) | 0.399 |
| Residence | Urban | 119(31.48%) | 43(11.41%) | <0.001 |
|  | Rural | 259(68.52%) | 334(88.59%) |  |
| Educational status | Primary (1-8) | 181(47.88%) | 172(45.62%) | <0.001 |
|  | Secondary & above | 49(12.96%) | 20(5.31%) |  |
|  | Read & write | 43(11.38 %) | 12(3.18%) |  |
|  | Not literate | 105(27.78%) | 173(45.89%) |  |
| Religion | Orthodox Christian | 61(16.14%) | 22(5.84%) | <0.001 |
|  | Islam | 290(76.72%) | 341(90.45%) |  |
|  | Others | 27(7.14%) | 14(3.71 %) |  |
| Ethnicity | Gurage | 284(75.13%) | 234(62.07%) | <0.001 |
|  | Silte | 71(18.78%) | 94(24.93%) |  |
|  | Other | 23(6.08%) | 49(13.00%) |  |
| Occupational status | Farmer & Housewife | 31(8.20%) | 47(12.47%) | 0.240 |
|  | Housewife | 296(78.31%) | 287(76.13%) |  |
|  | Merchant | 34(8.99%) | 30(7.96%) |  |
|  | Other | 17(4.50%) | 13(3.45%) |  |
| Marital status | Currently married | 376(99.47) | 376(99.73%) | 0.564 |
| SES, wealth indices | Poorest | 46(12.37%) | 100(27.70%) | <0.001 |
|  | Poor | 63(16.94%) | 84(23.27%) |  |
|  | Middle | 76(20.43%) | 69(19.11%) |  |
|  | Rich | 79(21.24%) | 69(19.11%) |  |
|  | Richest | 108(29.03%) | 39(10.80%) |  |
| MUAC | Mean (SD) | 24.83(2.26) | 24.60(2.06) | 0.166 |
| Hgb | Mean (SD) | 13.13(1.19) | 13.03(1.20) | 0.241 |
| BMI | Mean (SD) | 21.59(3.10) | 21.62(6.00) | 0.915 |
| GA | Mean (SD) | 16.74(4.52) | 16.70(4.61) | 0.910 |
| LTE | Mean (SD) | 0.57(1.15) | 1.17(1.43) | <0.001 |
| Perceived stress | Mean (SD) | 16.09(3.36) | 16.48 (6.84) | 0.320 |
| State-trait anxiety | Mean (SD) | 12.32(3.22) | 13.84(5.30) | <0.001 |
| PRA score | Mean (SD) | 24.41(10.96) | 26.76(9.53) | 0.002 |
| MSS score | Mean (SD) | 21.84(4.65) | 21.80(4.55) | 0.922 |
| IPV score | Mean (SD) | 4.63(1.54) | 4.77(1.36) | 0.166 |
| PHQ-9 at T1 | Mean (SD) | 2.50(3.37) | 4.82(4.26) | <0.001 |
| PHQ-9 at T2 | Mean (SD) | 2.22(3.16) | 4.69(4.60) | <0.001 |
| Baseline depression | Not depressed | 292 (77.25%) | 199 (52.79%) | <0.001 |
|  | Depressed | 86 (22.75%) | 178 (47.21%) |  |
| Perinatal depression | Not depressed | 200 (79.68%) | 156 (57.78%) | <0.001 |
|  | Depressed | 51 (20.32%) | 114 (42.22%) |  |
| SES, socio-economic status; MUAC, mid-upper arm circumference; Hgb, hemoglobin; BMI, body mass index; GA, gestational age; LTE, list of threatening experience; PRA, pregnancy-related anxiety; MSS, maternity social support; IPV, intimate partner violence; PHQ, patient health questionnaire  Data are presented as numbers (percent) unless otherwise specified. | | | | |

| **Supplementary Table 2: Path coefficients between predictors and endogenous variables in the path model** | | |
| --- | --- | --- |
| **Variable** | **Standardized coefficient (95% CI)** | ***p*-value** |
| Perinatal depression |  |  |
| State-trait anxiety | 0.15(0.066 0.230) | <0.001 |
| Baseline depression | 0.36(0.284 0.441) | <0.001 |
| Food insecurity | 0.11(0.031 0.190) | 0.007 |
| State-trait anxiety |  |  |
| Perceived stress | 0.67(0.627 0.0.719) | <0.001 |
| Food insecurity | 0.20(0.146 0.263) | <0.001 |
| Baseline depression |  |  |
| Perceived stress | 0.33(0.261 0.406) | <0.001 |
| IPV | 0.19(0.108 0.270) | <0.001 |
| Food insecurity | 0.25(0.167 0.338) | <0.001 |
| Perceived stress |  |  |
| IPV | 0.20(0.122 0.287) | <0.001 |
| Food insecurity | 0.087(0.004 0.171) | 0.041 |
| IPV |  |  |
| Food insecurity | 0.10(0.016 0.186) | 0.020 |
| Endogenous variables: IPV, intimate partner violence; perceived stress; baseline depression; state-trait anxiety, perinatal depression.  Exogenous variable: Food insecurity | | |
